# Sudden unilateral vision loss in an adult with Behcet disease: A case report

**DOI:** 10.1097/MD.0000000000013594

**Published:** 2018-12-14

**Authors:** Yunxia Gao, Shuyuan Lyu, Li Bao, Ming Zhang

**Affiliations:** Department of Ophthalmology, Sichuan University West China Hospital, Sichuan, China.

**Keywords:** Behcet disease, central retinal artery occlusion, central retinal vein occlusion, diagnosis, therapy, uveitis, vasculitis

## Abstract

**Rationale:**

Behcet disease is an autoimmune vasculitis, involving multiple systems and organs. It is often chronic and recurrent, and involves all levels of arteries and veins.

**Patient concerns:**

A 40-year-old Chinese male with sudden vision loss in his right eye for 3 days was reported.

**Diagnoses:**

The patient was diagnosed as bilateral uveitis (obstructive retinal vasculitis in the right eye) and Behcet disease.

**Interventions:**

The patient was given topical corticosteroids and compound tropicamide eye drops in our department. In addition, he was also hospitalized in the Rheumatology Department, and received systemic infusion of methylprednisolone and cyclophosphamide.

**Outcomes:**

Best corrected visual acuity vision acuity of the left eye was 20/20 and that of the right eye was no light perception after 2 weeks. During the telephonic follow-up after 1 year, the patient reported 2 recurrent episodes of uveitis in the left eye, which improved after treatment at the local hospital.

**Lessons:**

It cautions ophthalmologists of the high possibility of vasculitis in young and middle-aged patients with retinal vascular occlusion. It also reminds the ophthalmologists investigate systemic diseases, and obtain detailed medical history, not just depending on specific laboratory findings. This case further suggests that Behcet disease has diverse manifestations, complex diagnosis, and severe lesions, and the recurrence rate must be controlled to minimize irreversible consequences such as sudden vision loss during recurrence.

## 1 Introduction

Behcet disease is an autoimmune vasculitis, involving multiple systems and organs. It is often chronic and recurrent, and involves all levels of arteries and veins, thus leading to vascular occlusion.^[1]^ Patients with Behcet disease usually have eye involvement, most commonly uveitis. According to the characteristics, uveitis related to Behcet disease can be most manifested with acute nongranulomatous anterior uveitis, intermediate uveitis, obstructive or nonobstructive retinal vasculitis, focal or multifocal retinitis, and necrotizing retinitis, of which acute nongranulomatous anterior uveitis and obstructive retinal vasculitis are the most common.^[2,3]^ In obstructive retinal vasculitis, the blocking sites are often located in the branch retinal veins, and cases with central artery and vein occlusion are rare, in which patients show rapid decline or even loss of vision and have a very poor prognosis.^[2,4]^ This study reported a Behcet disease patient with sudden unilateral vision loss due to central retinal artery and vein occlusion.

## 2 Case report

**Figure 1:**
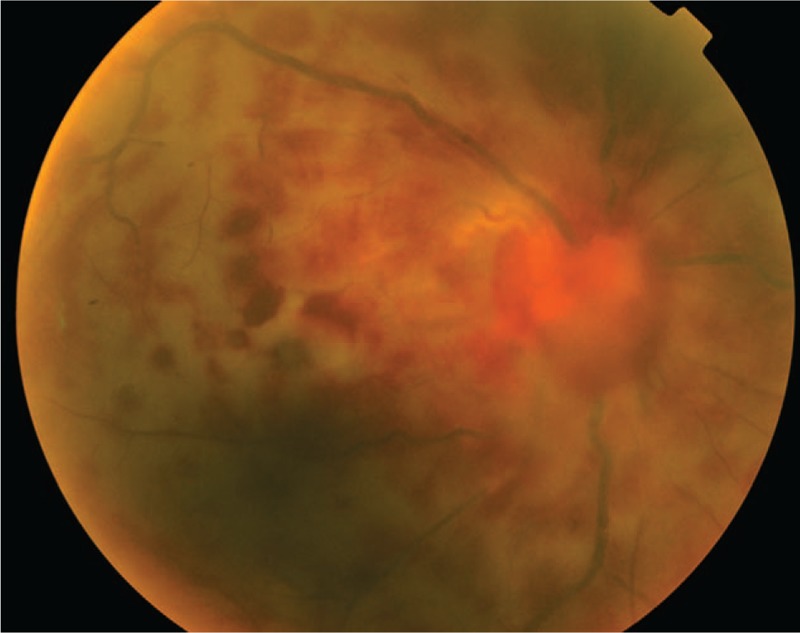
Fundus photograph (FP) of the right eye revealed that the retina was gray, with edema as well as scattered dotted and flaky hemorrhagic foci.

**Figure 2:**
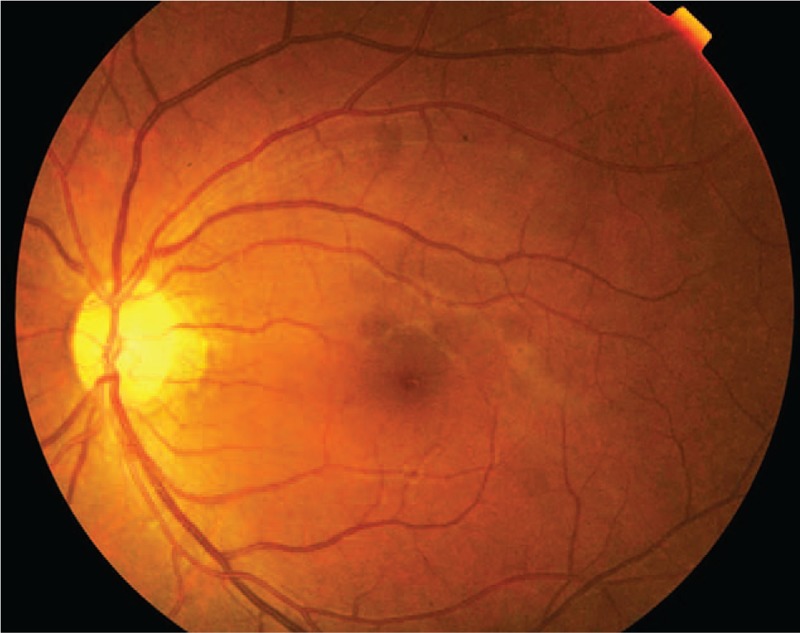
Fundus photograph (FP) of the left eye showed no obvious abnormal findings in the fundus.

**Figure 3:**
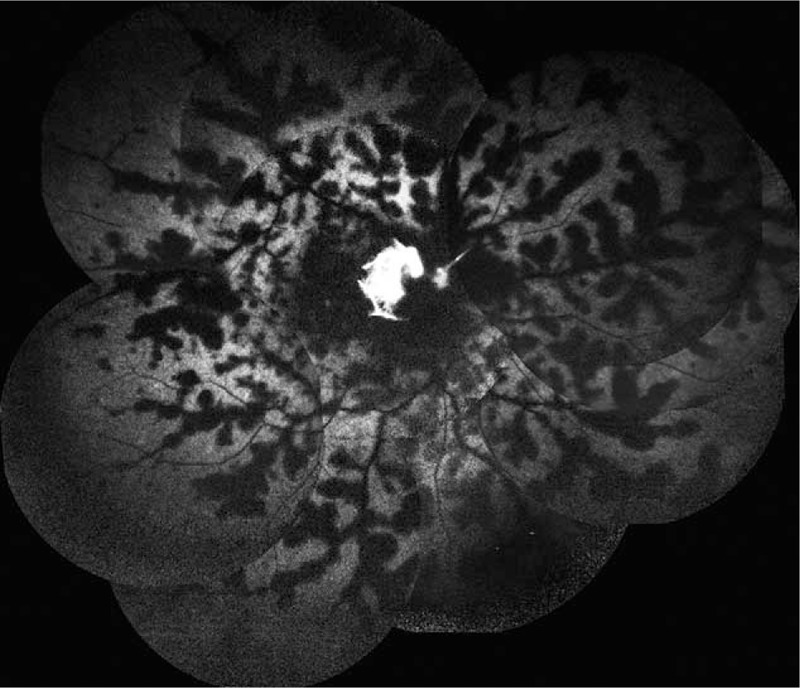
Fluorescein fundus angiography (FFA) of the right eye showed perfusion in retinal arteries near the optic disc, nonperfusion in other retinal blood vessels, hemorrhage along the retinal veins, which blocked the fluorescence in the mid-term stage of angiography.

**Figure 4:**
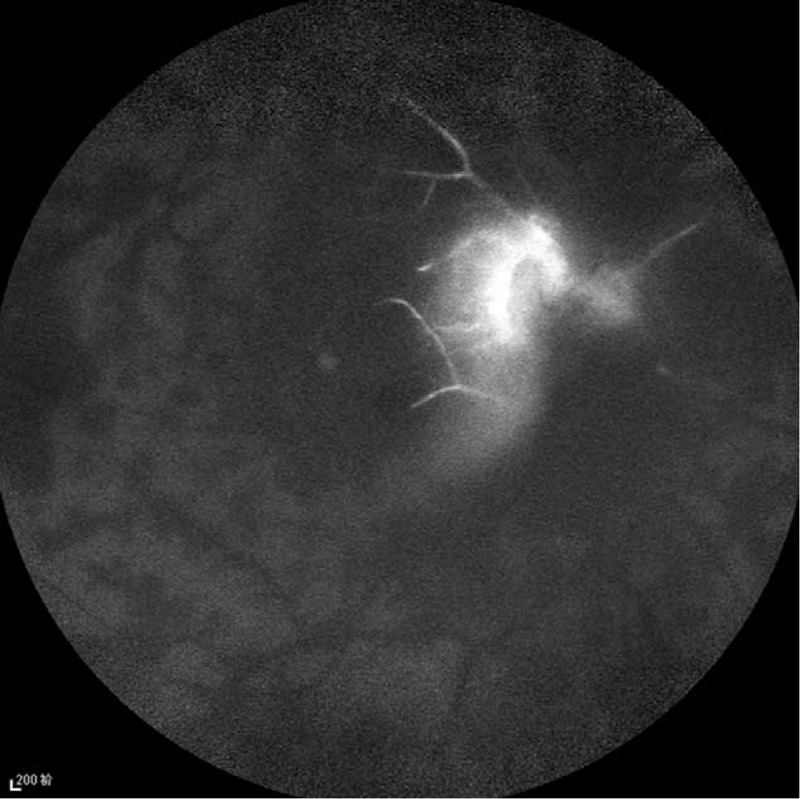
Fluorescein fundus angiography (FFA) of the right eye showed hyperfluorescence in the optic disc, perfusion still only in retinal arteries near the optic disc, but not in other retinal blood vessels in the late stage of angiography.

**Figure 5:**
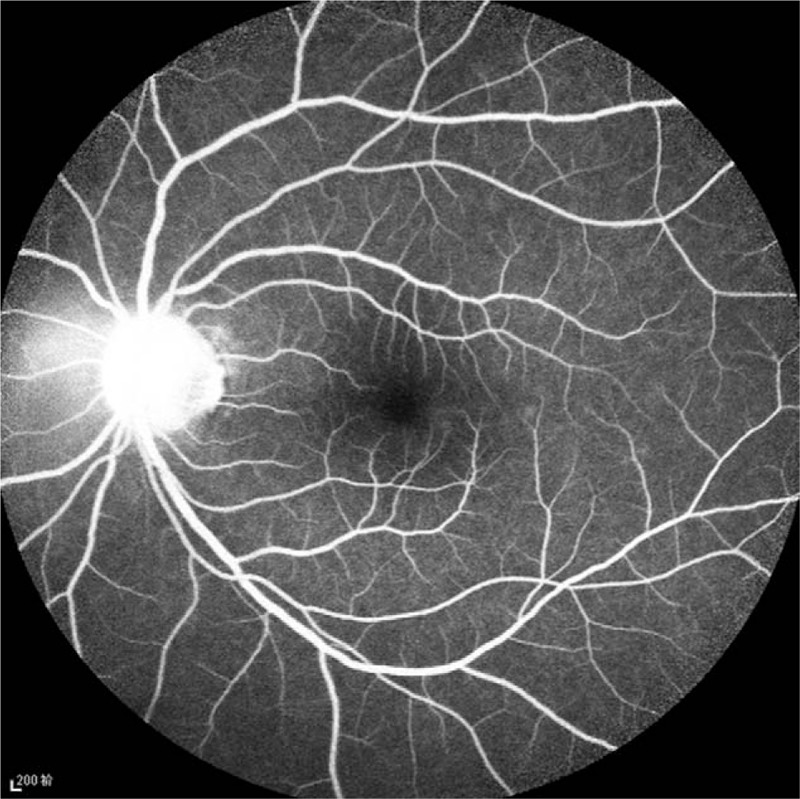
Fluorescein fundus angiography (FFA) of the left eye, telangiectasia was visible in the optic disc, with leakage in the disc in the stage of angiography.

A 40-year-old Chinese male presented with a 3-day history of sudden vision loss in his right eye. He complained of a sudden onset foreign body sensation in the right eye when he was riding, and vision loss after rubbing. Eye examination showed best corrected visual acuity was no light perception (NLP) in the right eye and 20/40 in the left eye. Anterior segment examination of the right eye showed keratin precipitates+, aqueous flare++, and vitreous opacity. The right fundus examination revealed that the retina was gray, with edema as well as scattered dotted and flaky hemorrhagic foci (Fig. 1). In addition, hyalocytes were visible in the left eye, without obvious abnormalities in the fundus (Fig. 2). Fluorescein fundus angiography showed the following results: For the right eye, the arm-to-retinal circulation time was 19.54″, background fluorescence of the choroid in the early angiography was not uniform. At 21.10", the optic disc showed hyperfluorescence, with perfusion in some disc blood vessels. Until the late stage of angiography at 10′30.20″, perfusion was seen only in retinal arteries near the optic disc, but not in other retinal blood vessels. The optic disc showed hyperfluorescence, and hemorrhage was visible along the retinal veins, which blocked the fluorescence (Figs. 3 and 4). For the left eye, telangiectasia was visible in the optic disc, with leakage in the disc in the late stage of angiography (Fig. 5). Auxiliary examination showed that erythrocyte sedimentation rate (ESR) was 50 mm/h and C-reactive protein (CRP) was 18 mg/L. There were no significant abnormalities in blood routine, antineutrophil cytoplasmic antibodies, anticardiolipin antibody, direct antiglobulin testing, *Toxoplasma gondii* infection, rubella virus, cytomegalovirus, and herpes simplex virus and immune examinations. Echocardiography, carotid duplex ultrasound, abdominal Doppler ultrasound, and chest computed tomography showed negative results. Past medical history revealed that the patient had complaints of recurrent oral ulcers and vulvar ulcers for 8 years, which recently worsened. The patient was diagnosed as Behcet disease at a local hospital and given long-term low-dose oral prednisone, but the recurrent symptoms persisted with a recent relapse. At our hospital, the patient was diagnosed as bilateral uveitis (obstructive retinal vasculitis in the right eye), and Behcet disease. He was given topical corticosteroids, and compound Tropicamide eye drops. The patient was also hospitalized in the Rheumatology Department, and received systemic infusion of methylprednisolone and cyclophosphamide. Two weeks later, his vision acuity of the left eye was 20/20 and that of the right eye was NLP. During the telephonic follow-up after 1 year, the patient reported 2 recurrent episodes of uveitis in the left eye, which improved after treatment at the local hospital.

## 3 Discussion

The typical clinical findings of Behcet disease are complex, including oral ulcer, vulvar ulcer, skin rash, erythema nodosum, uveitis, esophageal ulcer, colon ulcer, joint pain, etc. The diagnosis depends on typical clinical findings, without specific laboratory findings. Increase in CRP and ESR are probably the only laboratory findings.^[5]^ The etiology and pathogenesis of this disease is not fully understood. Vasculitis is the foremost histopathological lesion affecting various blood vessels in Behcet disease. According to vessel involvement, Behcet disease has demonstrated 3 main clinical features: venous occlusion, arterial occlusion, and aneurysm formation.^[6,7]^ Endothelial dysfunction leaded by vascular inflammation is thought to be a major factor for thrombosis.^[8]^ Thrombosis is the most important cause of vascular occlusion. Some studies have suggested that risk factors for thrombosis include increased endothelial activation markers, defective fibrinolysis, and/or impaired coagulation, reflect vascular cell injury, inherited thrombophilia, demographic, genes, immune system, race, HLA-B5 antigen, etc.^[9,10]^

Uveitis in patients with Behcet disease can affect the anterior segment, posterior, or peripheral retina. Patients with involvement of anterior segment usually develop nongranulomatous uveitis, with anterior chamber empyema being the most common. Meanwhile, patients with involvement of posterior segment usually develop vitreous opacity, retinal invasion, vascular sheaths, vascular occlusion, macular edema, and other inflammatory manifestations.^[11]^

For ocular lesions caused by Behcet disease, in addition to eye topical anti-inflammatory symptomatic treatment, corticosteroids are the most widely used systemic treatment, which combined with immunosuppressive agents can achieve better curative effect in patients with severe first episode or repeated relapse, and help to reduce drug side effects with a combination of glucocorticoids. Biological agents (such as tumor necrosis factor-blockers and alpha-interferon), which have been used in recent years, are expensive, and have several side effects and high relapse rates after discontinuation. Their safety, efficacy, and cost-effectiveness need further evaluation.^[11,12]^

Behcet disease has unclear etiology, and lacks characteristic laboratory findings. Its diagnosis is mainly based on clinical manifestations, and more positive signs lead to more reliable diagnosis. Hence, ophthalmologists should not exclude this systemic disease by solely relying on the laboratory findings of patients with uveitis. Missed diagnosis of Behcet disease is likely if the general clinical manifestations are ignored, thereby missing the optimal timing of treatment. Patients with Behcet disease are susceptible to various types of ocular inflammation with different manifestations. However, the patient in this study suffered from vascular occlusion and sudden vision loss because his unilateral retinal vasculitis affected both the central retinal artery and vein, which is very rare, and shows very poor curative effect and prognosis. This case also cautions ophthalmologists of the high possibility of vasculitis (such as Behcet disease) in young and middle-aged patients with retinal vascular occlusion. Hence, the ophthalmologists should investigate systemic diseases, and obtain detailed medical history. This case further suggests that Behcet disease has diverse manifestations, complex diagnosis, and severe lesions, and the recurrence rate must be controlled to minimize irreversible consequences such as sudden vision loss during recurrence.

Written informed consent was obtained for the publication of the case details.

## Acknowledgments

The authors want to thank Ms Xiaoyue Wang and Mr Zhibing Zeng in Imaging Department, Sichuan University West China Hospital for the excellent work on fundus photography and fluorescein fundus angiography.

## Author contributions

**Conceptualization:** Yunxia Gao, Ming Zhang.

**Data curation:** Yunxia Gao, Shuyuan Lyu, Li Bao.

**Formal analysis:** Yunxia Gao, Shuyuan Lyu.

**Investigation:** Yunxia Gao, Shuyuan Lyu, Ming Zhang.

**Methodology:** Yunxia Gao.

**Project administration:** Yunxia Gao, Li Bao, Ming Zhang.

**Resources:** Yunxia Gao, Li Bao, Ming Zhang.

**Supervision:** Yunxia Gao, Shuyuan Lyu, Ming Zhang.

**Writing – original draft:** Yunxia Gao.

**Writing – review and editing:** Yunxia Gao, Shuyuan Lyu, Ming Zhang.
